# The first complete chloroplast genome of *Adiantum nelumboides* (Pteridaceae), a rare and endangered fern species

**DOI:** 10.1080/23802359.2020.1711821

**Published:** 2020-01-16

**Authors:** Hongmei Liu, Yunjuan Zuo, Pingshan Zhan, Yu Shi

**Affiliations:** a Xishuangbanna Tropical Botanical Garden, Chinese Academy of Sciences, Menglun, China;; b Center for Integrative Conservation, Xishuangbanna Tropical Botanical Garden, Chinese Academy of Sciences, Menglun, China;; c School of Agriculture and Forest, Pu’er University, Pu’er, China

**Keywords:** Conservation genetics, rare and endangered species, medicinal plants, ornamental plants

## Abstract

The complete chloroplast genome of a rare and endangered fern species *Adiantum nelumboides* was sequenced. The genome has a typical quadripartite structure with a length of 149,956 bp and 42.8% GC content. We annotated totally 131 genes, including 87 protein genes, 36 tRNA, and 8 rRNAs. This work provides crucial information for its phylogenetic and conservation of a critically endangered fern and its value as an ornamental and medicinal plant resource.

*Adiantum nelumboides* X. C. Zhang is an epithilial fern belonging to the subfamily Vittarioideae of Pteridaceae (Zhang 2012; PPGI 2016). This species is a unique and beautiful plant with orbicular or orbicular-reniform single pinnule. *Adiantum nelumboides* is endemic to the Shizhu County (Sichuan, China) and is endangered in its native habitat by over-collecting, road-building, and water reservoir construction (Liao et al. 2007, 2008; Liu et al. 2007), and was categorized as critically endangered in China (Dong et al. 2017). This species is known as ‘He ye jin qian cao’ which has been used in Chinese medicine for more than 100 years. *Adiantum nelumboides* was once treated as *Adiantum reniforme* Linnaeus var. *sinense* Y. X. Lin (Lin 1980; Lin and Gilbert 2013), but it is now recognized as an independent species rather than a variety of *A*. *reniforme* (Zhang 2012). The status was supported by cytological, palynological, and molecular phylogenetic evidence (Wang et al. 2015). Because of its unique orbicular-reniform pinnule shape, it is now cultivated as an ornamental plant.

In this study, we reported the complete chloroplast genome of *A*. *nelumboides* for the first time. Fresh leaf material was collected from Xishuangbanna Tropical Botanical Garden, Chinese Academy of Sciences, Yunnan, China, and the voucher specimen was deposited at the Herbarium of Xishuangbanna Tropical Botanical Garden (HITBC, voucher number: Liu-CP12). The experiment procedure including genome sequencing, assembly, and annotation is as described in Liu et al. (2019). Previously published plastomes from the same genus, including *A. aleuticum*, *A. capillus-veneris*, *A. shastense*, and *A. tricholepis* were used to guide the sequence assembling and annotation. The newly sequenced plastid genome was submitted to the GenBank (accession number MN709399).

The complete chloroplast genome of *A*. *nelumboides* is 149,956 bp in length with 42.8% GC content, with the typical quadripartite structure including a large single-copy (LSC) region of 82,936 bp and a small single-copy (SSC) region of 21,483 bp, which are separated by two inverted repeats (IR) of 22,596 bp. The plastome consists of 131 genes (87 protein-coding genes, 36 tRNA, and 8 rRNA genes).

The phylogenetic position of *A*. *nelumboides* was reconstructed by incorporating the newly generated plastome of *A*. *nelumboides* into a matrix including 29 taxa covering all five subfamilies of Pteridaceae (PPGI 2016), with two species of subfamily Cryptogrammoideae (*Cryptogramma acrostichoides* and *Llavea cordifolia*) designed to be the outgroup taxa. 84 coding genes were selected, assembled, and aligned into a single matrix. The phylogenetic analyses were carried out using RAxML with 1000 bootstrap replaced under the GTRGAMMAI substitution model (Stamatakis 2006).

Five species of *Adiantum* formed a clade, with *A. nelumboides* and *A. capillus-veneris* as sister to each other (Figure 1). Two subfamilies (Cheilanthoideae and Vittarioideae) are strongly supported as monophyletic; however, the subfamily Parkerioideae was founded to be clustered in the subfamily Pteridoideae.

**Figure 1.**
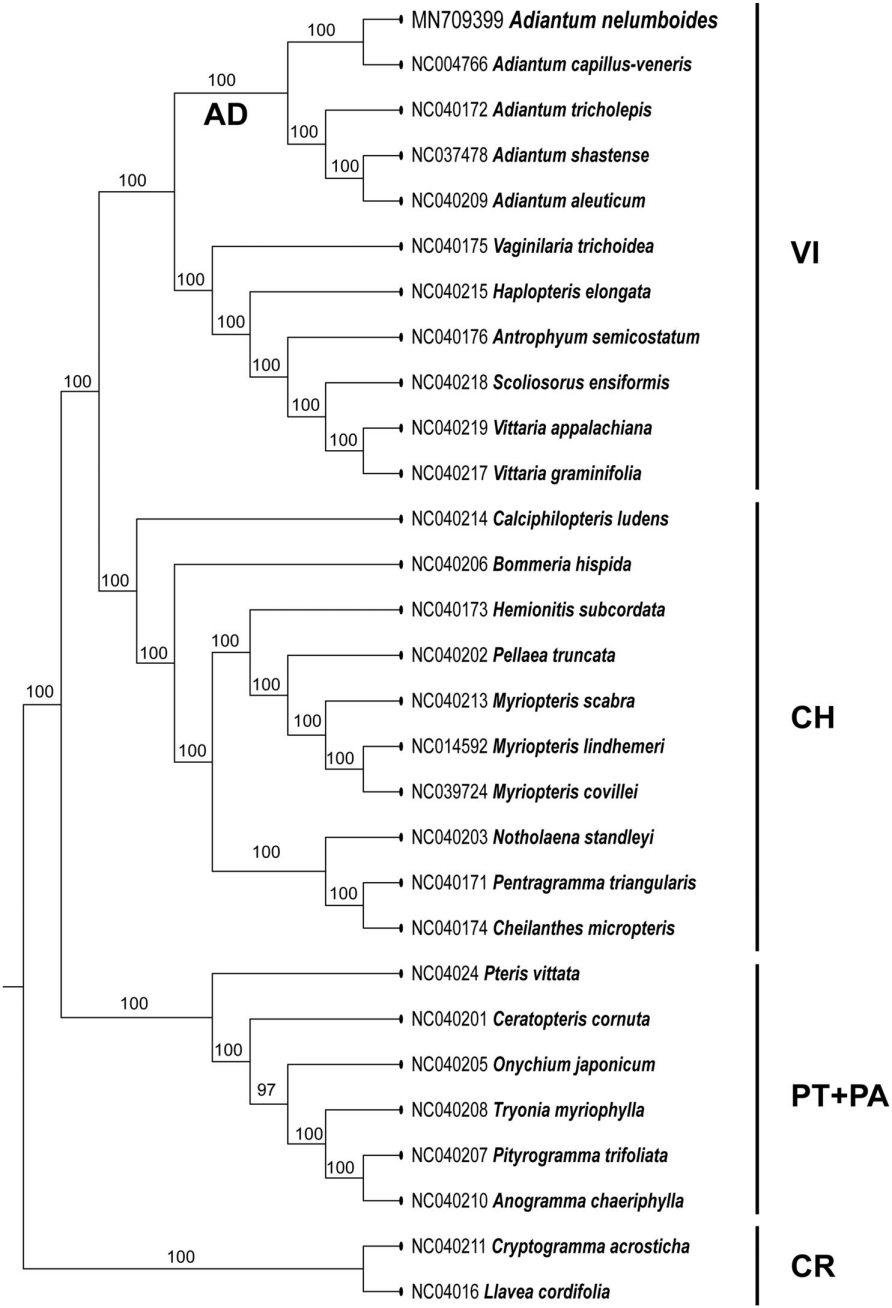
Maximum likelihood phylogeny recovered from 29 chloroplast genomes by RAxML. The sampling included representatives of all five subfamilies of Pteridaceae. Two species of the subfamily Cryptogrammoideae were selected as outgroup. AD: *Adiantum*; CH: Cheilanthoideae; CR: Cryptogrammoideae; PA: Parkerioideae; PT: Pteridoideae; VI: Vittarioideae.

The complete chloroplast genome sequence will provide important and useful resources extending conservation genetics (Liu et al. 2007; Fu and Chen, 2019) of this ornamental and medicinal species, especially to the ex*-situ* conservation of this critical endangered species (Huang et al. 2008; Liao et al. 2013; Wu et al. 2010).
